# Academic psychological capital questionnaire 12 (APCQ-12): psychometric validity and measurement invariance in a Chilean sample of high school students

**DOI:** 10.3389/fpsyg.2023.1229170

**Published:** 2023-11-21

**Authors:** Marcos Carmona-Halty, Geraldy Sepúlveda-Páez, Carla Semir-González, Karina Alarcón-Castillo, Patricio Mena-Chamorro

**Affiliations:** Escuela de Psicología y Filosofía, Universidad de Tarapacá, Arica, Chile

**Keywords:** psychological capital, psychometric analyzes, high school students, invariance, academic PsyCap

## Abstract

The present study evaluated the psychometric properties of the Academic Psychological Capital Questionnaire 12 (APCQ-12) in a sample of 2,196 Chilean high school students (51% girls) aged 12 to 17 years (mean 14.83 years). Results showed that: (1) the APCQ-12 produces adequate scores in terms of reliability, (2) the internal structure of the questionnaire obtains adequate fit indices, for a second order model, which is consistent with previous research, and (3) the APCQ-12 proved to be sex and age invariant. Overall, the APCQ-12 proved to be an adequate questionnaire for measuring academic psychological capital in Chilean high school students, producing valid and reliable scores.

## Introduction

In recent years, psychological capital (PsyCap) –a core construct from positive organizational behavior research is integrated by four psychological resources: hope, self-efficacy, resilience, and optimism (acronym HERO resources)– has received increasing attention in the academic context given its relevance in predicting student performance and well-being (e.g., Datu et al., 2016; King and Caleon, 2021; Ramírez-Pérez, 2022; Tomás et al., 2022; Carmona-Halty et al., 2022a). Academic PsyCap describes those students who: 1) persevere in the pursuit of academic goals and, if necessary, redirect the pathways leading to the successful completion of those goals (i.e., are hopeful); 2) possess the confidence to take on challenging academic tasks and exert the effort necessary to succeed at them (i.e., are efficacious or self-efficacious); 3) tolerate and bounce back in adverse or problematic academic circumstances (i.e., they are resilient); and 4) make positive attributions related to past, current, and future academic success experiences (i.e., they are optimistic; Martínez et al., 2021).

Although the HERO resources have been shown individually to be relevant in predicting desirable academic outcomes (e.g., Snyder et al., 2002; Le Ouweneel et al., 2011; Gallagher et al., 2017; Oriol-Granado et al., 2017), the notion of simultaneity of PsyCap makes a difference, as a whole, as its components would act synergistically, conferring a more significant effect than would be achieved with the sum of their individual effects (Luthans et al., 2007). This notion is consistent with the Conservation of Resources (COR) theory, which points out that resources do not exist in isolation because people try to accumulate as many as possible in the form of resource caravan (Hobfoll, 1989, 2002). Accordingly, the academic PsyCap can be understood as a caravan of resources –composed of hope, efficacy, resilience, and optimism– that originates in the motivation of students to accumulate psychological resources that –as research has shown– promote the successful resolution of particular challenges, placing students in a unique vantage point to address new demands and challenges, which translates into increased levels of well-being and performance (Hobfoll, 1989, 2002; Hobfoll et al., 2018).

Previous research has shown that academic PsyCap, on one hand, is directly related to motivation (Datu et al., 2016), engagement (Carmona-Halty et al., 2021), coping strategies (Ramírez-Pérez, 2022), self-regulated learning (Sava et al., 2020), well-being (Poots and Cassidy, 2020; King and Caleon, 2021), and performance (Carmona-Halty et al., 2019; Luthans et al., 2022); and, on the other, it is inversely related to indicators of boredom (Kang et al., 2021), stress (Lisnyj et al., 2022; Xu and Wang, 2022), procrastination (Hicks and Wu, 2015), burnout (Carmona-Halty et al., 2022b), and depression (Finch et al., 2020; King and Caleon, 2021). The above suggests that academic PsyCap is a relevant variable in predicting optimal student functioning and a construct applicable to the goals of positive education, that is, the study of students’ academic skills and well-being (Seligman et al., 2009). The reason for this is that academic PsyCap functions as a high-order resource that regulates the behaviors and thoughts of students, providing vitality that stimulates their intrinsic motivation, keeping them engaged in academic pursuits, and enabling them to achieve their academic goals (Luthans and Youssef-Morgan, 2017; Li et al., 2023).

The most widely used instrument to assess PsyCap is the Psychological Capital Questionnaire (PCQ) developed by Luthans et al. (2007). Originally, this questionnaire included six items measuring each of the four HERO resources contained in the PsyCap construct. The items from PCQ-24 were selected from established measures –hope (Snyder et al., 1996), efficacy (Parker, 1998), resilience (Wagnild and Young, 1993), and optimism (Scheier and Carver, 1985)– and were wording adapted to the work conditions (Luthans and Youssef-Morgan, 2017). Its structure comprises one second-order factor (i.e., PsyCap) and four first-order factors (i.e., HERO resources). Later, for pragmatic reasons, Avey et al. (2011) validated a 12-item version of PCQ-24 (hope, four items; efficacy, three items; resilience, three items; and optimism, two items) showing that its structure is invariant across cultures, countries, and sex (e.g., Caza et al., 2010; Rus et al., 2012; Wernsing, 2014; Luthans and Youssef-Morgan, 2017). More recently, based on the premise that students are the future workers in the labor market, some researchers have been using the PCQ in the academic context (e.g., Luthans et al., 2012; Kang et al., 2021; King and Caleon, 2021). In this line, Martínez et al. (2021) adapted the PCQ-12 to the everyday conditions of Spanish-speaking undergraduate university students, calling it the Academic Psychological Capital Questionnaire 12 (APCQ-12). Specifically, using two samples of undergraduate university students (from Chile and Spain), they demonstrated that the APCQ-12 replicates the original structure of the PCQ-12, that this structure is invariant across cultures, and produces scores that are significantly related to students’ engagement (i.e., vigor, dedication, and absorption), satisfaction with study (i.e., with the university, the faculty to which they belonged, the program in which they were studying, and their professors), and academic performance (i.e., GPA score).

Since the appearance of the APCQ-12, it is possible to note a growing interest in Spanish-speaking countries in the study of academic PsyCap, preferentially among samples of undergraduate university students (e.g., Ortega-Maldonado and Salanova, 2018; Martínez et al., 2021; Sánchez-Cardona et al., 2021; Ramírez-Pérez, 2022; López-Guerra et al., 2023). Despite these valuable efforts, knowledge about PsyCap in Spanish-speaking countries is still in its infancy, and more research efforts are required to have conclusive results by considering other educational groups, such as secondary education (e.g., Schönfeld and Mesurado, 2020; Carmona-Halty et al., 2021; Tomás et al., 2022). In this line, to our knowledge, only two studies have addressed the psychometric properties of the APCQ-12 in a population of Spanish-speaking secondary students. First, Schönfeld and Mesurado (2020), using a sample of 313 Argentine students, report a four-related factor solution and significant relationships between PsyCap, engagement, and achievement. Second, Tomás et al. (2022), obtained evidence supporting the second-order structure using a sample of 267 Spanish students, comparing three competitive models (one factor, four related factors, and a second-order structure). Despite these studies’ contribution, none have measurement invariance; therefore, the analysis of the psychometric properties of the APCQ-12 in this population segment has been only partially addressed.

The present brief report examines the psychometric properties of the APCQ-12 and measurement invariance in a sample of Chilean high school students. More specifically, the reliability of the scores, evidence of internal structure, and measurement invariance across sex and age groups. This study is intended as a contribution to the applicability of the APCQ-12 in this population of students; future longitudinal studies that consider academic trajectories from school to the beginning of university or work life; the implementation of interventions aimed at promoting the components of the construct; and the implementation of studies in Spanish-speaking school contexts.

## Materials and methods

### Participants

A sample of 2,196 high school students (51% identified as girls) residing in two regions in the extreme north of Chile (i.e., Arica and Iquique) aged 12 to 17 years (M = 14.83 SD = 1.71) was achieved. They came from four public and private educational institutions, each one hosting approximately 650 students.

### Procedures

Participants were selected using a non-probability convenience sampling strategy (Otzen and Manterola, 2017). The procedure for collecting information consisted of contacting the directors through a letter of invitation explaining the study’s objectives. Once the participation of the educational establishments was accepted, parents or legal guardians were contacted. The aims and requests of the study were explained to each of them (through a consent form). Additionally, this procedure was replicated in an expository way to the students in the classrooms. Parents and students who decided to participate signed an informed consent and assent form, which explained that participation did not imply any compensation or incentive and that the data collected would be confidential and anonymous. The application was carried out in groups of 20 students during class hours and in the computer rooms of each establishment, where the students completed the questionnaire in an online format. The response procedure lasted approximately 10 min.

The Scientific Ethics Committee of the Universidad de Tarapacá approved the research.

### Instruments

The Academic Psychological Capital Questionnaire 12 (APCQ-12; Martínez et al., 2021) is a measure of self-report that jointly assesses levels of hope (e.g., “*I can think of many ways to reach my current goals regarding my studies*”), efficacy (e.g., “*I feel confident contributing to discussions about strategies on my studies*”), resilience (e.g., “*I usually take stressful things in stride concerning my studies*”), and optimism (e.g., “*I am optimistic about what will happen to me in the future as it pertains to my studies*”). It has a structure of four first-order factors (i.e., HERO resources) and 1 second-order factor (i.e., academic PsyCap). It uses a 6-point Likert scale as a response option, from 1 “*strongly disagree*” to 6 “*strongly agree*.”

The APCQ-12 items were tested on a small sample of secondary school students (n = 12) prior to general data collection to determine if there were any comprehension difficulties; at this stage, none of the students reported difficulties in understanding the items. Sampling was by convenience and non-probability (Ato et al., 2013); none of the participants were removed from the dataset.

### Data analysis

A confirmatory factor analysis (CFA) was performed with the statistical program Mplus, version 8.2 (Muthén and Muthén, 1998) to establish evidence of validity based on the internal structure of the test. For this, the maximum likelihood estimation method with standard errors and a mean and variance adjusted chi-square test statistic (MLMV) was used, as the data do not fit a normal distribution (the Shapiro–Wilk test showed evidence that the normality assumption *p* < 0.001 was not satisfied). The global fit indicators of the models were interpreted according to the guidelines proposed by Hair et al. (2019). For models with sample sizes greater than 250 participants and with fewer than 12 observed variables, values greater than 0.96 on the Comparative Fit Index (CFI) or Tucker-Lewis Index (TLI) and values less than 0.07 on the Root Mean Square Error of Approximation (RMSEA) when the CFI is equal to or greater than 0.96 were interpreted as adequate fit indicators. It should be remarked that the Standardized Root Mean Square Residual (SRMR) indicator was not used because it is usually biased under these conditions. Additionally, suppose the model presents indicators of poor fit or any non-significant parameter. In that case the model will be re-specified based on the content of the items and the modification indexes iteratively. Furthermore, to establish the equivalence of the APCQ-12 between students’ sex and age, 2 second-order multigroup CFA models were performed, following the recommendations of Wang and Wang (2019) for estimating these models. A difference of 0.010 or less in the comparative fit index (ΔCFI) was considered to indicate measurement invariance (Cheung and Rensvold, 2002; Chen, 2007; Dimitrov, 2010). Reliability and the corrected homogeneity index were estimated for each dimension by means of Cronbach’s alpha and McDonald’s hierarchical omega coefficients in their non-ordinal versions using the Jamovi statistical program (The Jamovi Project, 2021) In the same way, the composite reliability coefficient was estimated considering values above 0.60 for acceptable reliability (Diamantopoulos et al., 2000). Finally, the average variance extracted was calculated as a criterion of evidence of convergent validity (considering acceptable values above 0.50) (Henseler et al., 2009). Also, discriminant validity was assessed by comparing the square root of the AVE and Pearson’s correlation coefficient. Independence between the factors was fulfilled when the square root of the AVE exceeded the correlation between the factors (Fornell and Larcker, 1981).

## Results

### Preliminary analysis and reliably of the scores

The descriptive statistics for each item that makes up the APCQ-12 are presented in Table 1. According to the univariate normality test, the items do not follow a normal distribution (Aldás and Uriel, 2017). As for the reliability estimates presented, these were adequate both for the general factor (*α* = 0.90; *ω* = 0.90) and for each of its dimensions: hope (*α* = 0.83; *ω* = 0.84), efficacy (*α* = 0.79; *ω* = 0.79), resilience (*α* = 0.72; *ω* = 0.73), and optimism (*α* = 0.76; *ω* = 0.76) (Cho and Kim, 2015). Additionally, the corrected results of the homogeneity index suggest that no item should be deleted.

**Table 1:** Descriptive and reliability coefficients (Cronbach’s alpha and McDonald’s omega) for each item.

	Descriptive statistics				Reliability statistics		
	M (SD)	S	K	W	CHI	*α* if item is dropped	*ω* if item is dropped
1. Efficacy	4.08 (1.20)	−6.30	−3.27	0.925*	0.616	0.901	0.903
2. Efficacy	3.98 (1.25)	−4.71	−4.66	0.930*	0.639	0.900	0.902
3. Efficacy	4.07 (1.31)	−8.61	−4.28	0.921*	0.576	0.903	0.905
4. Hope	4.29 (1.26)	−9.78	−3.33	0.912*	0.668	0.898	0.901
5. Hope	3.58 (1.30)	−3.31	−5.23	0.935*	0.671	0.898	0.901
6. Hope	4.14 (1.19)	−6.28	−3.65	0.924*	0.705	0.897	0.899
7. Hope	3.63 (1.33)	−3.34	−6.12	0.936*	0.686	0.898	0.900
8. Resilience	3.99 (1.35)	−6.37	−5.85	0.927*	0.553	0.904	0.906
9. Resilience	3.43 (1.47)	−1.24	−9.00	0.931*	0.554	0.905	0.906
10. Resilience	3.98 (1.22)	−5.78	−3.44	0.928*	0.683	0.898	0.900
11. Optimism	3.92 (1.34)	−6.07	−5.34	0.930*	0.651	0.899	0.902
12. Optimism	4.27 (1.26)	−11.31	−0.77	0.909*	0.671	0.898	0.901
Academic PsyCap	3.95 (0.90)	−2.01	−2.78	0.995*	–	0.908	0.909

* *p* < 0.001; SD, Standard Deviation; S, Skewness standardized; K, Kurtosis standardized; W, Shapiro–Wilk test; CHI, corrected homogeneity index; M, Media.

The results of composite reliability showed values consistent with the recommendations (i.e., adequate values above 0.60) (Diamantopoulos et al., 2000) for the HERO dimensions and as well as for the overall PsyCap measure. As for the average variance extracted, the values obtained for each latent variable were above 0.5, as recommended (Henseler et al., 2009), thus supporting the convergent validity of the model, except for the resilience dimension. See Tables 2, 3 for details. The results of evidence discriminant validity indicate that it is possible to sustain evidence of discriminant validity (*r* = 0.532–0.661; the square roots of AVE = 0.663–0.783).

**Table 2:** Composite reliability and average variance extracted for the APCQ-12.

Latent variable	Composite reliability (p_c_)	Average variance extracted (p_v_)
Efficacy	0.826	0.614
Hope	0.826	0.574
Optimism	0.766	0.524
Resilience	0.611	0.440
PsyCap	0.944	0.585

p_c_, Composite Reliability; p_v_, Average variance extracted.

**Table 3:** Correlations between PsyCap factors.

Variable	*r*			
	Hope	Efficacy	Resilience	Optimism
Hope	0.757^1^			
Efficacy	0.639*	0.783^1^		
Resilience	0.659*	0.532*	0.723^1^	
Optimism	0.661*	0.538*	0.626*	0.663^1^

* *p* < 0.001; ^1^ Square to AVE.

### Sources of validity evidence of internal structure

**Figure 1:**
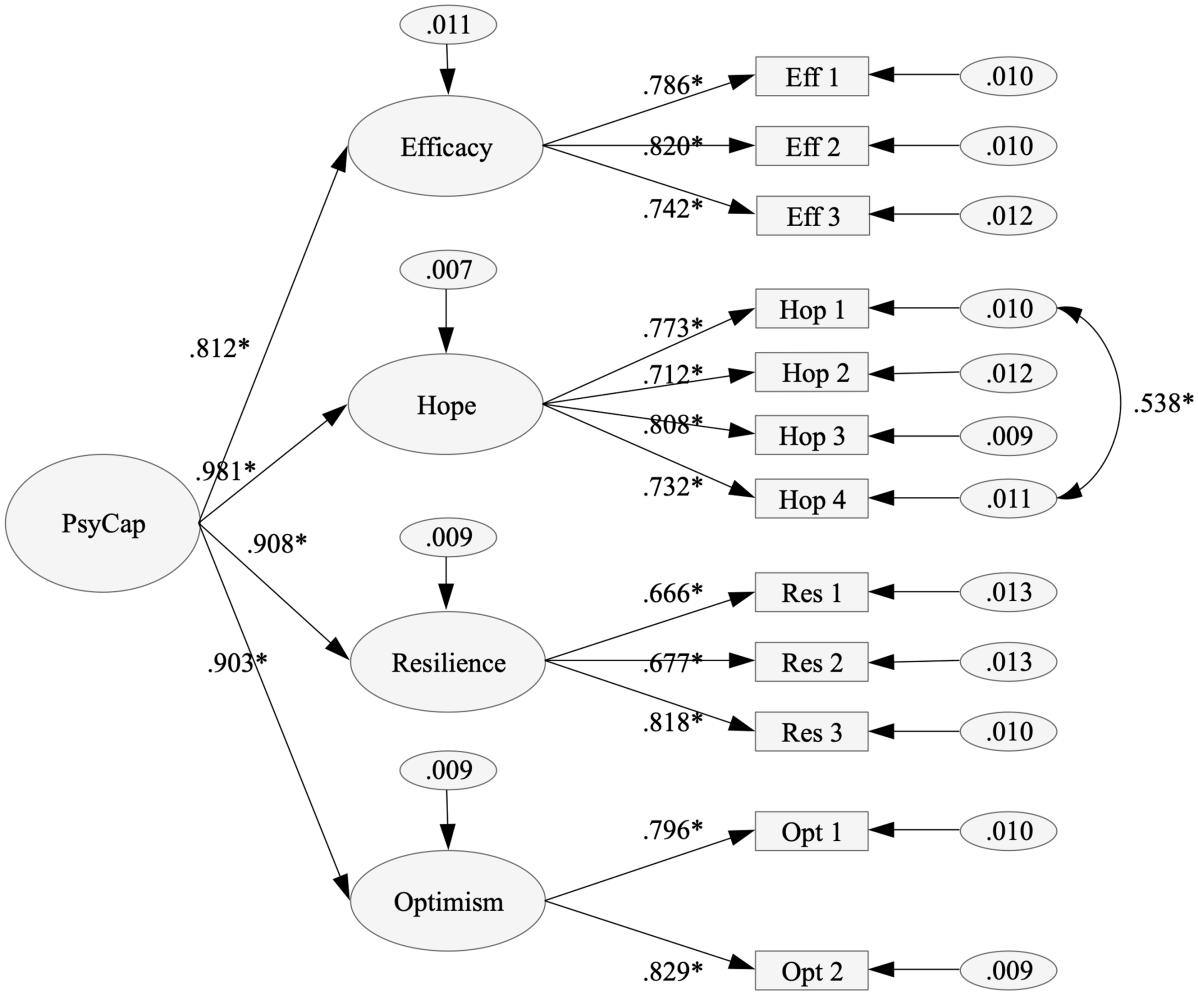
Measurement model of APCQ-12.

The internal structure of the instrument was assessed by estimating a CFA model with four first-order factors (i.e., HERO) and a second-order factor (M1). The results of the CFA analysis indicated that the model did not sufficiently explain the observed covariation matrix (see Table 4). Consequently, the model was re-specified –based on the modification indices and the item content, which refers to a favorable assessment of the student’s current state concerning studies– by covarying the error variances between items 4 (“*I am currently being quite successful in my studies*”) and 7 (“*At the moment I am achieving the goals I have set for myself as a student*”) of the hope dimension. As a result, the re-specified version of the model denoted as M1a in Table 4, demonstrated an adequate fit to the data. Figure 1 shows the factor loadings obtained for the M1a model. In this case, high factor loadings are observed in the first-order structure (*λ* > 0.50), with a notable representation of the second-order factor in the Standardized beta coefficients (*β* > 0.50).

**Table 4:** Fit indexes for single–group and multiple–group CFA of the Academic Psychological Capital Questionnaire 12.

	*χ* ^2^	*df*	*p*	*χ*^2^/*df*	Δ*χ*^2^	Δ*df*	Δ*p*	RMSEA	90% CI	CFI	TLI	SRMR	CMs	ΔCFI
Single–group CFA														
M1 Second order	1348.198	50	0.000	26.963	–	–	–	0.109	[0.104, 0.114]	0.961	0.949	0.031	–	–
M1a Second order revised	520.451	49	0.000	10.621	–	–	–	0.066	[0.061, 0.071]	0.986	0.981	0.020	–	–
Sex invariance														
M2. Configural	244.254	94	0.000	2.598	–	–	–	0.038	[0.032, 0.044]	0.983	0.977	0.026	–	–
M3. Metric	258.452	102	0.000	2.533	13.700	8	0.089	0.037	[0.032, 0.043]	0.983	0.978	0.028	M2–M3	0.000
M4. Scalar	334.290	110	0.000	3.039	98.967	8	0.000	0.043	[0.038, 0.048]	0.975	0.970	0.033	M2–M4	0.008
M5. Configural*	313.505	102	0.000	2.093	–	–	–	0.043	[0.038, 0.049]	0.977	0.970	0.038	–	–
M6. Metric*	299.569	109	0.000	2.748	86.064	7	0.000	0.040	[0.035, 0.045]	0.979	0.975	0.032	M5-M6	0.002
Age invariance														
M7. Configural	333.884	188	0.000	1.775	–	–	–	0.044	[0.037, 0.052]	0.977	0.968	0.034	–	–
M8. Metric	364.775	212	0.000	1.720	30.134	24	0.180	0.043	[0.035, 0.050]	0.976	0.971	0.042	M7–M8	0.001
M9. Scalar	408.424	236	0.000	1.730	79.643	48	0.002	0.043	[0.036, 0.050]	0.973	0.970	0.044	M7–M9	0.004
M10. Configural*	386.470	204	0.000	1.894	–	–	–	0.048	[0.040, 0.055]	0.972	0.963	0.044	–	–
M11. Metric*	476.792	265	0.000	1.799	95	61	0.008	0.059	[0.053, 0.065]	0.963	0.963	0.058	M10–M11	0.003

*, second order invariance; χ^2^, Chi-square; df, degree of freedom; RMSEA, Root Mean Square Error of Approximation; 90% CI, Confidence Interval; CFI, Comparative Fit Index; TLI, Tucker-Lewis Index; SRMR, Standardized Root Mean Square Residual; CMs, Comparisons between models; Δ *χ*^2^, The difference in model *χ*^2^ statistics between the models; Δ CFI, CFI differential.

### Measurement invariance across sex and age

Two second-order multigroup CFA models were performed to assess the measurement invariance of the APCQ-12 between sex and age groups. First, second-order configurational invariance (unrestricted model) was estimated. The results show that the configural model, for sex and age, fits the data adequately (see Table 4, M5 and M10), allowing it to be used as a benchmark for comparing more restrictive models. Second, to assess the invariance of the factor loadings in a second-order model, it is necessary to estimate the invariance in the first-order factors preliminarily. Table 4 shows the results of the metric and scalar invariance analysis of the first-order factors, both for sex (M2-M4) and age (M7-M9). In both cases, the CFI differences were less than 0.010, suggesting that the models show metric and scalar equivalence. Third, after confirming invariance in the first-order factor loadings and item intercepts, we assessed invariance in the second-order factor loadings by verifying whether the relationships between the four first-order factors (i.e., HERO resources) and the academic PsyCap are consistent across groups. The model fits were adequate, and the differences in CFI met the established criteria, supporting the invariance of the second-order factor loadings of the PsyCap between the groups according to sex and age.

## Discussion

This study aimed to examine the psychometric properties of the Academic Psychological Capital Questionnaire 12 (APCQ-12) and measurement invariance in a Chilean high school student sample.

Firstly, the results indicate that the APCQ-12 presents adequate levels of internal consistency for each of its dimensions (i.e., hope, efficacy, resilience, and optimism) and for the general factor (PsyCap). Additionally, the corrected homogeneity index suggests that no item needed to be removed. Also, the APCQ-12 showed satisfactory evidence of convergent and discriminant validity. These findings support the accuracy and reliability of the scale, which is consistent with the reliability reported in previous studies using the PCQ-12 and the APCQ-12 (León-Pérez et al., 2016; Martínez et al., 2019, 2021). Second, regarding the estimation of the factor model, the results evidenced that the structure of the PsyCap, respecified, adequately fit the data. These support the relevance and applicability of the instrument (PsyCap) in Chilean high school students, in line with prior research (León-Pérez et al., 2016; Martínez et al., 2021; Tomás et al., 2022). Third, the second-order multigroup CFA evidenced that it is possible to sustain measurement invariance. Therefore, the model would be appropriate for Chilean high school students and comparable for boys and girls and across age groups. Although no theoretical reasons are mentioned in the literature of PsyCap for assuming that the APCQ-12 is variable among boys and girls of different ages, we consider it is relevant to show that this evidence is consistent with studies conducted in academic and workplace contexts (Wernsing, 2014; Kang et al., 2021; López-Guerra et al., 2023).

The main strength of the present study is the large sample used. However, there also some limitations should be considered when interpreting the results. First, it is essential to mention that this study did not examine the concurrent validity of the APCQ-12. Therefore, further research is needed to evaluate this psychometric property and reinforce the evidence found in this study. Second, a non-probability sampling was used, which limit the generalization of the results to the Chilean student population. Therefore, it would be necessary to carry out future studies using probability sampling to verify the instrument’s structure in a more representative sample of the Chilean student population. Third, the information collected in this study was self-reported, which may increase the likelihood of common method variance or other biases such as social desirability. Therefore, it would be advisable for future research to include other sources of information, either external peer or teacher evaluations, and thus provide a more complete understanding of the psychometric properties of the APCQ-12.

Despite these limitations, this study represents a significant contribution since it provides evidence of the psychometric properties of the APCQ-12 in a sample of Chilean high school students, which broadens the understanding and use of this tool in the educational context. This contribution is particularly relevant since the PsyCap has been related to various aspects of student’s psychological well-being and performance (e.g., Datu et al., 2016; King and Caleon, 2021; Ramírez-Pérez, 2022; Tomás et al., 2022; Carmona-Halty et al., 2022a). Furthermore, the study supports using a brief instrument, which may facilitate student participation.

## Data availability statement

The raw data supporting the conclusions of this article will be made available by the authors, without undue reservation.

## Ethics statement

The studies involving humans were approved by Comité Ético Científico Universidad de Tarapacá. The studies were conducted in accordance with the local legislation and institutional requirements. Written informed consent for participation in this study was provided by the participants' legal guardians/next of kin.

## Author contributions

All authors contributed equally to the research design and wrote the manuscript.
